# Patient-reported outcome domains for the esophageal CONDUIT report card: a prospective trial to establish domains

**DOI:** 10.1186/s12955-018-1023-7

**Published:** 2018-10-10

**Authors:** Minji K. Lee, Kathleen J. Yost, Karlyn E. Pierson, Shanda H. Blackmon

**Affiliations:** 1 Robert D. and Patricia E. Kern Center for the Science of Health Care Delivery, Mayo Clinic, Rochester, MN USA; 2 Department of Health Sciences Research, Mayo Clinic, Rochester, MN USA; 3 Department of Surgery, Mayo Clinic, 200 First St SW, Rochester, MN 55905 USA

**Keywords:** Dumping, Dysphagia, Esophageal cancer, Esophagus, Patient-reported outcome measures, Postoperative pain, Psychometrics, Questionnaires, Reflux, Stricture

## Abstract

**Background:**

Health-related quality of life (QoL) deteriorates immediately after esophagectomy. Patients may benefit from periodic assessments to detect increased morbidity on the basis of subjective self-reports. Using input from patients and health care providers, we developed a brief prototype for the esophageal conduit questionnaire (Mayo Clinic Esophageal **C**onduit Outcomes Noting Dysphagia/Dumping, and Unknown outcomes with Intermittent symptoms over Time after esophageal reconstruction [CONDUIT] Report Card) and previously used it in comparative research. The present study aimed to expand its content and establish health-related QoL and symptom domains of a patient-reported postesophagectomy conduit evaluation tool.

**Methods:**

We expanded tool content by selecting items measuring patient-reported symptoms from existing questionnaires or written de novo. A multidisciplinary group of clinician content-matter experts approved the draft tool, together with a designated patient advocate. The expanded tool was administered to patients postesophagectomy from March 1 to November 30, 2016. We established domains of conduit performance for score reporting through data analysis with exploratory factor analyses. We assessed psychometric properties such as dimensionality, internal consistency, and inter-item correlations in each domain and compared content coverage with other existing measures intended for this patient population. For data that were missing less than 50% of patient responses, the missing values were imputed.

**Results:**

Five multi-item domains were established from data of 76 patients surveyed after esophagectomy; single items were used to assess stricture and conduit emptying. For every multi-item domain, dominance of 1 factor was present. Internal consistency reliability estimates for the domains were 0.87, 0.78, 0.75, 0.80, and 0.83 and average inter-item correlations were 0.40, 0.50, 0.40, 0.33, and 0.73 for dysphagia, reflux, dumping-gastrointestinal symptoms, dumping-hypoglycemia, and pain, respectively. Some items observed to have lower inter-item correlation were reworded or flagged for removal at future validation. For reflux and dumping-related hypoglycemia, additional items were written after these analyses.

**Conclusions:**

The CONDUIT Report Card is a novel questionnaire for assessing QoL and symptoms of patients after esophageal reconstruction. It covers major symptoms of these patients and has good content validity and psychometric properties. The tool can be used to help direct patient care, guide intervention, and compare efficacy of different treatment options.

**Trial registration:**

ClinicalTrials.gov identifier No. 02530983 on 8/18/2015.

## Background

Complex and extensive surgical procedures on the esophagus are sometimes necessary to treat esophageal cancer, Barrett esophagus, or severe achalasia. The most common indication for an esophagectomy is esophageal cancer, which remains the eighth leading cause of cancer-related death in the world [1]. Esophageal cancer has the fastest growing incidence of any cancer in the United States [2], with an increase of 50% in the past 2 decades. Esophagectomy can offer about a 30% chance of cure in early-stage esophageal cancer and a 5-year overall survival rate of approximately 20% to 40% [3–9], with a risk of severe complications for approximately 40% of patients, as well as a recurrence rate of 32% to 54% in the first postoperative year [3–9].

Health-related quality of life (QoL) deteriorate immediately after esophagectomy, with patients having various symptoms within 6 months that include fatigue, insomnia, dry mouth, appetite loss, eating problems, dysphagia, reflux, esophageal pain, diarrhea, dyspnea, cough, and decreased social function [1, 10, 11]. Poor scores in QoL assessed at 6 months after esophageal cancer surgery were associated with an increased risk of death [11]. While patient-reported outcomes (PROs) have been increasingly endorsed by agencies and societies, they are not typically included in routine patient care [12]. For improvement in QoL and survival after esophagectomy, a patient may benefit from periodic assessments to detect increased morbidity on the basis of the patient’s subjective self-reports. After identification of eligibility for assessment through routine monitoring, the patient can be directed to symptom-specific interventions to better manage symptoms, to educate, and to improve health-related QoL.

Each of the existing patient-reported outcome instruments for esophagogastric cancer operations [13–19] has limitations. Cancer-specific PRO measures widely used after esophageal reconstruction that have generic and disease-specific components include the Functional Assessment of Cancer Therapy (FACT) and European Organization for Research and Treatment of Cancer (EORTC) questionnaires [19]. The 17–item Esophageal Cancer Subscale (ECS) can be combined with the total FACT-General score to produce a single FACT-E score [15, 16]. While both FACT-E and EORTC QLQ-OES18(cancer quality of life questionnaire for esophagus) questionnaires are designed to be specific to esophageal cancer, they do not capture the most pressing concerns of patients surgically treated with conduit reconstruction. For example, we found from our content analyses that neither addresses dumping syndrome. In addition, while the QLQ-OES18 measures reflux, it does so with only two questions, which may not adequately cover the content for that concern. Some cancer-specific PRO measures are designed to assess only postoperative disease-specific dysfunction following esophagogastric surgeries—Dysfunction After Upper Gastrointestinal Surgery (DAUGS20) [18] and Esophagus and Stomach Surgery Symptom Scale (ES) [17]. DAUGS20 contains 20 items measuring limited activity due to decreased food consumption, reflux, dumping, nausea and vomiting, deglutition difficulty, pain, difficulty in stool formation and passage, which gives a single score. DAUGS20 only reports one overall score rather than separate scores for different dimensions. ES provides four scores on cervico-thoracic symptoms (CTS), abdominal hypersensitivity symptoms (AHS), abdominal distension symptoms (ADS), and diet-induced systemic symptoms (DIS). However, the ES is more anatomically-based rather than symptom-based, and some symptoms such as ADS are not anticipated to be common concerns in patients following esophageal reconstruction.

We sought to create a multi-item questionnaire with greater content coverage that also provides separate domain scores on the major symptoms of patients with a reconstructed esophagus. Continuous score scales from a multi-item questionnaire enable more reliable reporting of current status and change. The questionnaire whose use is validated for assessing post-esophagectomy symptoms can facilitate comparisons of the effect of the different kinds of esophageal reconstruction (eg, type of conduit, presence of pyloric drainage, route of conduit, size of conduit) on health-related QoL, survival, and toxicities. The questionnaire may also allow for primary care providers to have greater insight into the management of these complex concerns and to ensure appropriate and cost-effective intervention. We previously developed a brief assessment tool or a prototype using single items to assess 5 symptoms, and we used it to compare postoperative outcomes of 2 surgical techniques [19]. We present the expanded content of the Mayo Clinic Esophageal Conduit Outcomes Noting Dysphagia/Dumping, and Unknown Outcomes With Intermittent Symptoms Over Time After Esophageal Reconstruction (CONDUIT) Report Card questionnaire and describe how we established domains for score reporting.

## Methods

### Questionnaire content

We used multiple sources to inform the content of the original questionnaire. From a previous study performed January 1, 2009, through December 31, 2013 [20], 432 postoperative patient encounters from a symptom management esophageal support group in Houston, Texas provided qualitative and quantitative data, informing the tool content. There were regular meetings and monthly encounters. The regular meetings included patients, caregivers, and family members. The monthly encounters included patients who had undergone esophagectomy and reconstruction. At these meetings, data were collected to record frequency and severity of symptoms of patients after esophageal reconstruction in a tabulated form. Discussions were led by a surgeon (S.H.B.) and another staff member trained in qualitative methods. The complaint or symptom was recorded by one provider as another provider served as the moderator. These data were reviewed by a multidisciplinary group of gastroenterologists, surgeons, internists, medical oncologists, radiation oncologists, nursing staff, and radiologists. Each member was asked to provide input and suggest additional symptoms, concerns, or severity scores that were applicable. This input was used to create a prototype of the CONDUIT Report Card survey [20], comprising five domains (dysphagia, GERD, dumping, pain, and physical activity) measured using single items with ordinal response scales. It was also used to compare outcomes among 45 non–Mayo Clinic patients who received 1 of 2 types of surgical procedures involving “supercharged” jejunal interposition (ie, a microvascular anastomosis to augment the blood supply to a conduit) versus a gastric conduit [20]. The prototype was pilot tested with 26 Mayo Clinic patients from August 1 through November 30, 2015 in Rochester, Minnesota. The value of this rudimentary version was development of major domains to evaluate our target population. However, single items can be limited in the ability to reliably capture information about a set of complex symptoms.

**Fig. 1:**
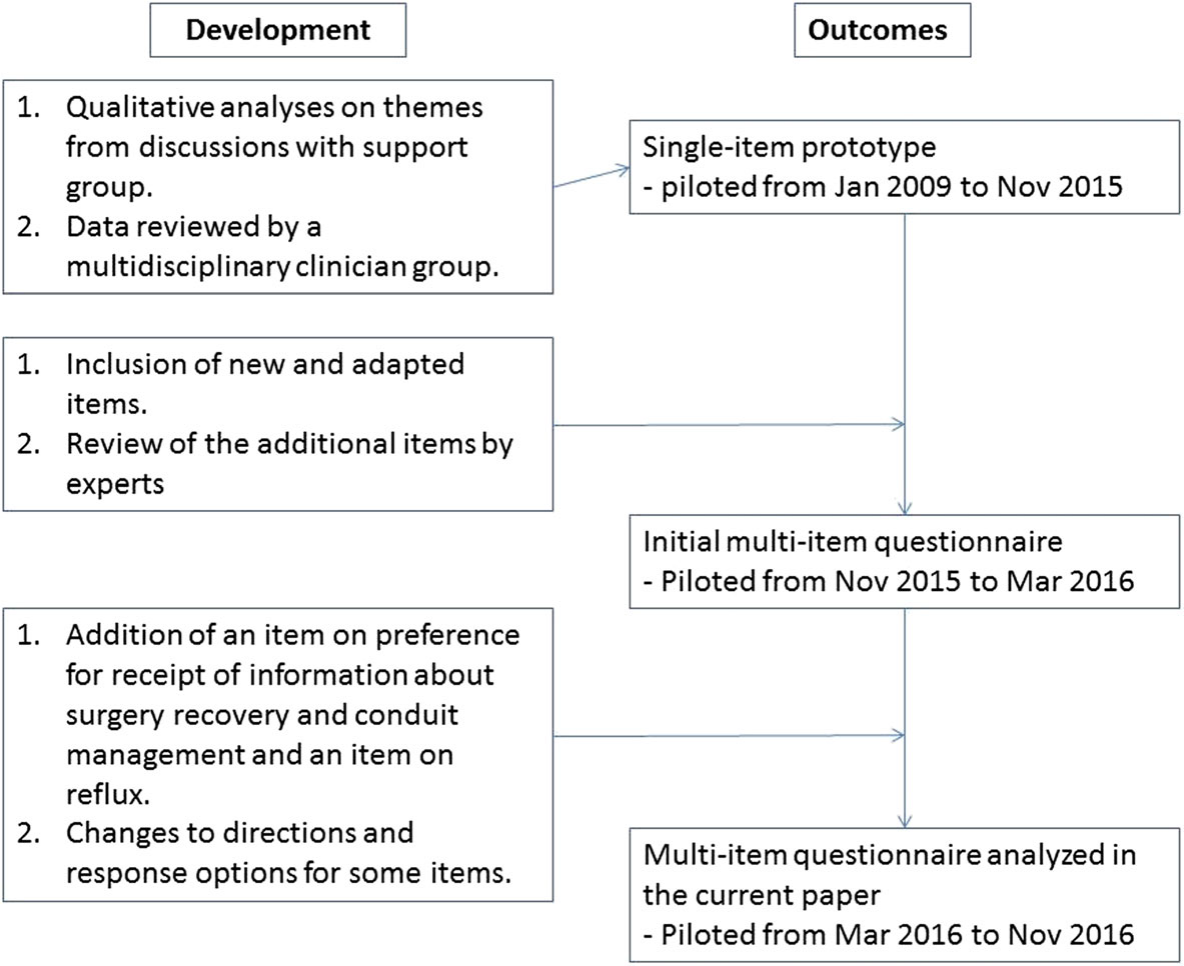
Development of the multi-item CONDUIT questionnaire

The trial was registered under ClinicalTrials.gov identifier No. 02530983 on 8/18/2015, where each patient who was administered the questionnaire at Mayo Clinic was prospectively enrolled and consented prior to administering the questionnaire. We expanded the prototype to have broader content coverage and include several multi-item scales. We created an item pool for each domain. Some items that measure symptoms were adapted (with permission) from Mayo Reflux Score [21], Modified Mayo Dysphagia Questionnaire–30 Day [22], and the scoring system for dumping syndrome by Sigstad [23]. A thorough evaluation of each item was performed by 2 clinician content-matter experts, a patient advocate, a survey design expert, and a psychometrician to assess the appropriateness of the questions for patients with esophageal reconstruction. We created a multi-item questionnaire, which was pilot tested with 45 Mayo Clinic patients from November 1, 2015, through February 29, 2016. Lastly, an item was added about the preference for receipt of information about surgery recovery and conduit management, as well as a reflux item rating the severity of acid regurgitation. Minor changes were made to instructions and response options for some items. Figure 1 describes the timeline of the development of the questionnaire and the dates associated with outcomes used to establish domains.

The current multi-item questionnaire, which is the subject of this manuscript, was administered to 78 Mayo Clinic patients from March through November 2016. We evaluated the dimensionality and the distribution of data collected with this version, which comprises sections on diet and swallowing, heartburn and acid regurgitation, and dumping syndrome. In addition, these patients were administered the following measures: health history, previous medical diagnoses, overall health and well-being as measured by Patient-Reported Outcomes Measurement Information System (PROMIS) Global Health short form [24], Eastern Cooperative Oncology Group (ECOG) performance status [25], and dyspnea as measured by the Medical Research Council breathlessness scale [26].

### Description of the sample

We analyzed the data of 76 unique consecutive patients, after the exclusion of data for 2 patients who sent in surveys with all responses missing. Among those with multiple observations, we selected one observation per patient by selecting the data with the least number of missing responses. If the number of missing responses was equal between observations, the tie breaker was to choose observations made at follow-up evaluation time points with the fewest total observations. Resulting data were composed of 6 surveys collected at 1 month after surgery, 10 at 3 months, 9 at 6 months, 11 at 9 months, 13 at 1 year, 20 between 2 to 5 years, and 7 with follow-up greater than 5 years.

### Establishment of domains to score patient-reported outcomes for conduit performance

#### Identification of domains and their indicators

We administered a validated QoL measure, the PROMIS Global Health-10 short form, which scores global physical health (GPH) and global mental health (GMH). The GPH portion rates ability to carry out every day physical activities, severity of pain, and severity of fatigue. The GMH part rates QoL related to mental health, including mood and the ability to think; satisfaction with social activities and relationships; and emotional problems. The raw scores (sums of item responses) on GPH and GMH were transformed manually to T-score metric using the look-up tables available from Health Measures [27]. For items borrowed or adapted, or both, from existing surveys [21–23], clinician content-matter experts identified the items associated with 7 themes: dysphagia, reflux, dumping, pain, lactose intolerance, stricture, and conduit emptying. For scoring, 14 items were associated with dysphagia, 8 with reflux, 16 with dumping, 2 with pain, 2 with lactose intolerance, and 1 each with stricture and conduit emptying. Many other additional items were intended to guide intervention but were not considered for computing scores, such as the specific food a patient has avoided or modified or the specific medication a patient takes to manage symptoms.

### Statistical analyses

We assessed dimensionality of the domains by examining the eigenvalue plots (scree plots) obtained from the item correlation matrix, internal consistency with the coefficient α [28], and inter-item correlations using the Kendall rank correlation coefficient within each of the multi-item domains. We multiply-imputed the missing values for cases with less than 50% of responses missing, performing the calculation separately for each domain. Before imputation, dimensionality was assessed with eigenvalue plots to make valid assumptions about the factor structure. When unidimensionality could be assumed, the missing values were imputed using item response models and taking the median of the 50 multiply-imputed datasets. To support score reporting for each domain, dominance of one factor was assessed with scree plots from exploratory factor analyses, using the principal axes method. The ratio of the first eigenvalue to the second, the location where the scree plot clearly levels off and the variability accounted for by the first factor were used to interpret the eigenvalues obtained from this analysis. Internal consistency reliability estimate was computed using coefficient α [28], of which the acceptable cutoff value was 0.70. Internal consistency describes the extent to which all the items in a test measure the same concept or construct. The coefficient α is grounded in the ‘tau equivalent’ model which assumes that each item measures the same latent construct [29]. If the scale has items with heterogeneous content, it would violate the assumption of tau equivalent model. Therefore, α is the lower bound estimate of reliability. Each item was correlated with another using Kendall’s rank correlation coefficient in its respective domain to determine whether any item had unusually low correlation with others. We computed raw scores for the resulting multi-item domains. For all newly developed domains, higher scores indicated greater symptoms. All analyses were performed using R software [30].

## Results

The 76 patients returned 118 surveys, with 28 providing their data twice and 7 providing the data 3 times at different points of follow-up. Age of patients at the time of surgery ranged from 24 to 89 years (mean, 66 years). Our sample was composed of 18 women and 58 men. Of the 76 patients, 72 were non-Hispanic white, and 1 was Hispanic or Latino. Four patients did not have cancer: 3 had end-stage achalasia and 1 had complications after hernia repair. Among the 72 cancer patients, 48 received chemoradiotherapy; 1, chemotherapy; 5, endoscopic mucosal resection; and 15, no neoadjuvant therapy before esophageal reconstruction. With respect to surgery types, 72 had esophagectomy, 2 had gastrectomy with reconstruction, and 2 had supercharged pedicled jejunal interpositions.

### PROMIS Global Health

The GPH and GMH domains had 4 items each. Ten observations had more than 50% of missing data for GPH and GMH and were removed from the analyses. In the current sample, GPH scores across all assessment time points ranged from 26.7 to 67.7 (mean [SD], 48.0 [8.4]). Patients whose measurements were taken at 1- or 3-month follow-up (*n* = 49) had a mean GPH score of 46.4 compared with 53.4 at 5-year follow-up or later (*n* = 12). In addition, GMH scores across all time points ranged from 25.1 to 67.6 (mean [SD], 49.9 [9.0]). The average of GMH scores at 1- or 3-month follow-up was 50.1 and at 5-year follow-up or later was 55.6. Within 3 months postoperatively, patients had slightly less physical and comparable mental health as the general public whose average score is 50.0 for GPH and GMH (SD of 10). Those who survived 5 years or longer after surgery did slightly better in both physical and mental health than the general public.

### Dysphagia

**Fig. 2:**
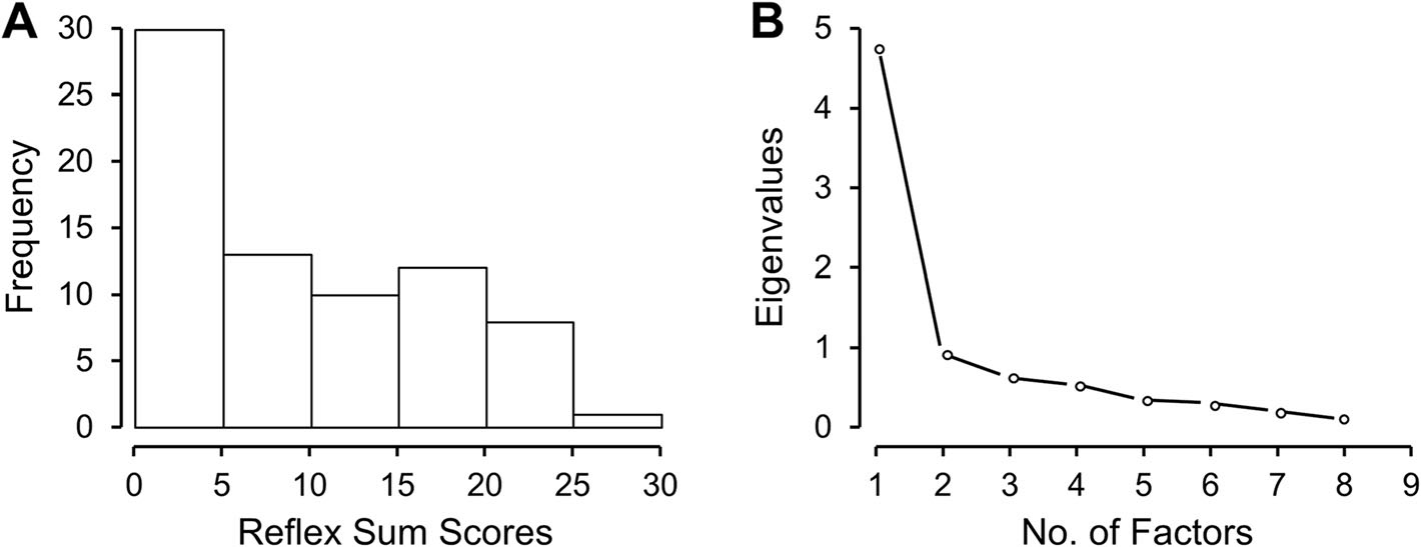
Histogram (**a**) and Scree Plot (**b**) of Dysphagia

Dysphagia items numbered 14; one observation with eight items missing was excluded. The other surveys had 1 missing response on average, which was imputed. The item statistics on the 75 observations are presented in Table 1. Forty-one patients reported no trouble swallowing. The summed score can range from 0 to 38. The scores of our sample were positively skewed, ranging from 0 to 27 (mean, 7.69; median, 3) (Fig. 2a).

**Table 1:** Dysphagia items with abbreviated stems and item statistics^a^

Item Stem	Response Categories	Item Statistics, Min, Median, Max; Mean (SD)
Frequency of difficulty swallowing	0 (never) to 4 (daily)	0, 0, 4; 1 (1.39)
Frequency of difficulty swallowing on one day	0 (never) to 2 (each swallow)	0, 0, 1; 0.45 (0.50)
Problems swallowing liquids	0 (no) and 1 (yes)	0, 0, 1; 0.13 (0.34)
Problems swallowing solid foods	0 (no) and 1 (yes)	0, 0, 2; 0.39 (0.52)
Severity of your trouble swallowing	0 (no trouble swallowing) to 4 (very severe)	0, 0, 4; 1.04 (1.31)
Severity of your trouble swallowing	0 (not at all severe) to 10 (very severe)	0, 0, 8; 1.43 (2.31)
Pills got stuck in esophagus/swallowing tube	0 (no) and 1 (yes)	0, 0, 1; 0.17 (0.38)
Solid food (not medications) got stuck in esophagus/swallowing tube	0 (no) and 1 (yes)	0, 0, 1; 0.28 (0.45)
Duration of time solid food was stuck	0 (not stuck) to 2 (≥5 min)	0, 0, 2; 0.35 (0.60)
Problems swallowing liquids after the solid food was stuck	0 (no) and 1 (yes)	0, 0, 1; 0.12 (0.33)
Solid food that got stuck made you vomit	0 (no) and 1 (yes)	0, 0, 1; 0.08 (0.27)
Pain or discomfort when swallowing	0 (does not hurt at all) to	0, 0, 2; 0.31 (0.52)
	2 (hurts all the way down most of the time when I swallow)	
Minutes taken to eat an average meal	0 (15 min) to 4 (> 60 min)	0, 1, 4; 0.95 (0.79)
Swallowing in general	0 (able to eat anything) to 4 (unable to swallow anything)	0, 1, 3; 1 (0.77)

Abbreviations: max, maximum; min, minimum; NA, not applicable

^a^Pilot items from CONDUIT Report Card; ©2018 Mayo Foundation for Medical Education and Research; all rights reserved

Dominance of one factor was present in that the plot levelled off after the first factor, the ratio of the first eigenvalue of 6.83 to the second eigenvalue of 1.72 was 3.97, and the variability accounted for by the first factor was 49.0% (Fig. 2b). The coefficient α for dysphagia was 0.87. The average inter-item correlation among all items to other items in this domain was at least 0.20 (range, 0.22–0.56) with one exception. The item “In the past 7 days, how many minutes did it take you to eat an average meal?” had an average inter-item correlation of 0.14 with the other items of dysphagia. This item became a candidate for deletion because speed of eating may be affected by both speed of swallowing and eating habits unrelated to conduit performance.

### Reflux

**Fig. 3:**
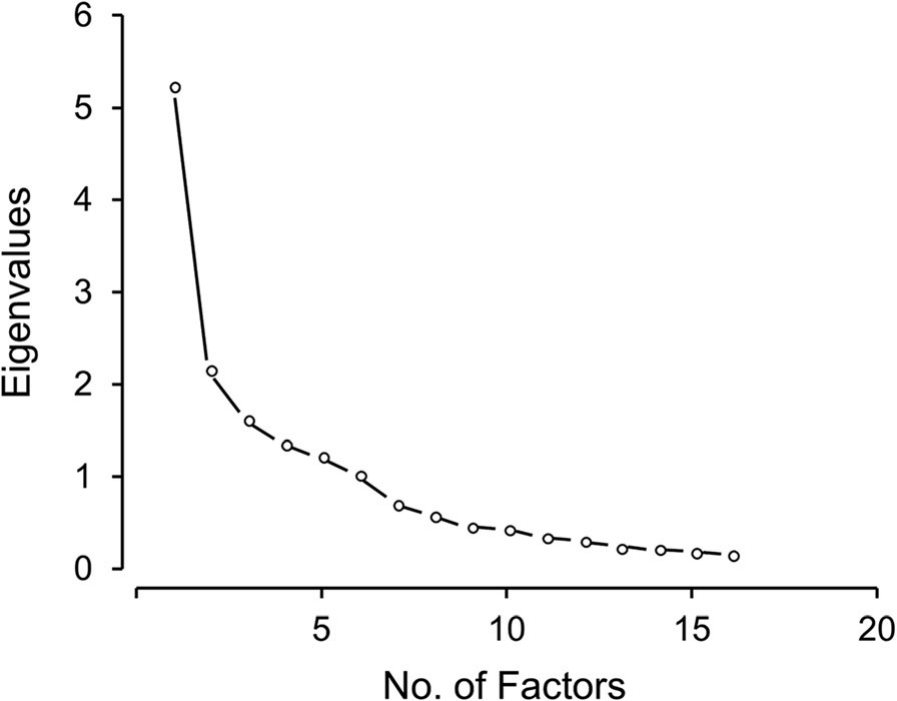
Histogram (**a**) and Scree Plot (**b**) of GERD. GERD indicates gastroesophageal reflux disease

In the tool, reflux is measured with questions assessing acid regurgitation (“bitter or sour-tasting fluid coming into your throat or mouth”) and heartburn (“a burning pain or discomfort behind the breastbone in your chest”). This scale comprises 8 reflux items. Two observations had more than 50% of missing responses—one with all items missing and one with five items missing—and were excluded. Item statistics of the reflux data are presented in Table 2. Twenty-eight patients reported no experience of heartburn, and 33 reported no acid regurgitation in the past 30 days. The possible summed score can range from 0 to 30. In our sample, scores were positively skewed, ranging from 0 to 27 (mean, 9.40; median, 7.5) (Fig. 3a).

**Table 2:** Acid reflux items with abbreviated stems and item statistics^a^

Item Stem	Response Categories	Item Statistics, Min, Median, Max; Mean (SD)
Use of antacids to manage heartburn	0 (no) and 1 (yes)	0, 0, 1; 0.45 (0.50)
Frequency of heartburn	0 (never) to 5 (daily)	0, 1, 5; 1.72 (1.75)
Severity of heartburn	0–10	0, 2, 8; 2.62 (2.84)
Waking at night due to heartburn	0 (no) and 1 (yes)	0, 0, 1; 0.34 (0.48)
Heartburn traveling up toward neck	0 (no) and 1 (yes)	0, 0, 1; 0.41 (0.49)
Had acid regurgitation	0 (no) and 1 (yes)	0, 1, 1; 0.55 (0.50)
Severity of acid regurgitation	0–10	0, 1, 10; 2.85 (3.26)
Use of medication to manage acid regurgitation	0 (no) and 1 (yes)	0, 0, 1; 0.47 (0.50)

*Abbreviations*: *max* maximum, *min* minimum

^a^Pilot items from CONDUIT Report Card; ©2018 Mayo Foundation for Medical Education and Research; all rights reserved

Dominance of one factor was shown because there was a clear elbow after the first eigenvalue, the ratio of the first eigenvalue (4.74) to the second eigenvalue (0.96) was 4.95, and variability accounted for by the first factor was 59.3% (Fig. 3b). The coefficient α for reflux was 0.78. Each item’s average inter-item correlation to other items was high, ranging from 0.36 to 0.72. To improve content coverage, we added two binary items measuring acid regurgitation: “In the past 30 days, has acid regurgitation caused your voice to become hoarse?” and “In the past 30 days, has acid regurgitation caused you to experience coughing?” We also added an item about aspiration, which can indicate severe reflux symptoms [31]. Aspiration was addressed as “In the past 7 days, how often have you inhaled food, drink, vomit or saliva into your lungs?”

### Dumping

**Fig. 4:**
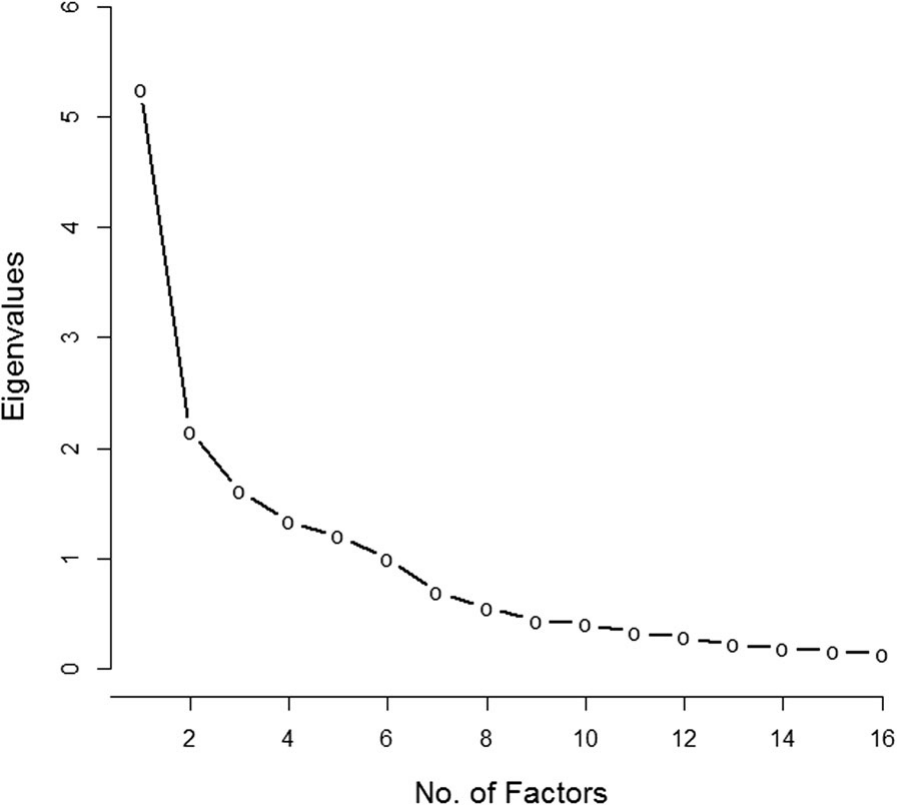
Scree Plot of Dumping

Dumping is defined in the CONDUIT tool as “Surgery to your abdomen can affect the size of your stomach and how it works. As a result, food may enter your small intestine faster and in larger amounts than it did before surgery. This may lead to *dumping syndrome*. Dumping syndrome is what the group of your symptoms is called. You may also hear it called *rapid gastric emptying*.” Seventeen items influenced dumping, and six observations had more than 50% of missing responses. One person had all items missing and five had at least 10 missing responses, which were excluded. Dimensionality of the 70 other surveys was investigated by computing eigenvalues of 16 items. The “fainting, loss of consciousness, passing out” item could not be included in this computation because no patients had this symptom, and the resulting covariance was zero between this item and all the other items. The scree plot from this analysis is presented in Fig. 4, which suggested more than 1 dimension.

The ratio of the first eigenvalue to the second eigenvalue was only 2.43, and the variability accounted for by the first factor was 32.8%. The scree plot started to level off after the second dimensions. Therefore, we conducted exploratory factor analysis with two factors using a cutoff of greater than the absolute value of 0.30 to identify items with salient factor loadings. The two factors were interpreted as measures of gastrointestinal (GI) tract symptoms and hypoglycemia (Table 3). The “vomiting, being sick to your stomach, throwing up, having dry heaves” item did not meet the 0.30 cutoff, was judged to be redundant with the “nausea or feeling like wanting to throw up” item, and was excluded from further analyses. The “sweating, feeling warmth, clammy” item loaded similarly on both GI tract symptoms and hypoglycemia, but the content of this item was deemed to be aligned more closely with hypoglycemia.

**Table 3:** Factor loadings from exploratory factor analysis with 2 factors for dumping syndrome items^a^

Item Stem	Factor 1^b^	Factor 2^b^
Shock	0.05	**0.35**
Fainting	Not applicable^c^	Not applicable^c^
Breathlessness	− 0.28	**0.96**
Weakness	−0.01	**0.85**
Sleepiness	0.15	**0.66**
Rapidly beating heart	0.01	**0.42**
Restlessness	0.06	**0.55**
Headaches	0.08	**0.39**
Sweating	**0.35**	**0.33**
Nausea	**0.36**	0.07
Abdominal fullness	**0.83**	0.00
Rumbling sound from stomach	**0.79**	−0.05
Belching	**0.47**	−0.02
Vomiting	0.21	0.04
Diarrhea	**0.90**	−0.17
Frequency of above symptoms	**0.77**	0.01
Symptoms with each meal	**0.51**	−0.15

^a^Pilot items from CONDUIT Report Card; Item stems are abbreviated. ©2018 Mayo Foundation for Medical Education and Research; all rights reserved

^b^Factor loadings > 0.30 are considered salient and are shown in bold type

^c^The “fainting, loss of consciousness, passing out” item was removed from analysis because no variability was observed in the answer among the sample

For missing values, we assumed 2 correlated factor structures between GI tract symptoms and hypoglycemia and took the median value of the 50 multiply-imputed datasets. Item statistics of the dumping data are presented in Table 4. Twenty-one patients reported no experience of dumping syndrome in the past 30 days.

**Table 4:** Dumping syndrome items with abbreviated stems and item statistics^a^

Item Stem	Response Categories	Item Statistics, Min, Median, Max; Mean (SD)
Hypoglycemia		
Shock	0 (no) and 1(yes)	0, 0, 1; 0.11 (0.32)
Fainting	0 (no) and 1 (yes)	0, 0, 0; 0.00 (0.00)^b^
Breathlessness	0 (no) and 1 (yes)	0, 0, 1; 0.24 (0.43)
Weakness	0 (no) and 1 (yes)	0, 0, 1; 0.39 (0.49)
Sleepiness	0 (no) and 1 (yes)	0, 0, 1; 0.37 (0.49)
Rapidly beating heart	0 (no) and 1 (yes)	0, 0, 1; 0.19 (0.39)
Restlessness	0 (no) and 1 (yes)	0, 0, 1; 0.23 (0.42)
Headaches	0 (no) and 1 (yes)	0, 0, 1; 0.09 (0.28)
Sweating	0 (no) and 1 (yes)	0, 0, 1; 0.34 (0.48)
Gastrointestinal Tract Symptoms		
Nausea	0 (no) and 1 (yes)	0, 0, 1; 0.37 (0.49)
Abnormal fullness	0 (no) and 1 (yes)	0, 1, 1; 0.60 (0.49)
Rumbling sound from stomach	0 (no) and 1 (yes)	0, 1, 1; 0.60 (0.49)
Belching	0 (no) and 1 (yes)	0, 1, 1; 0.57 (0.50)
Diarrhea	0 (no) and 1 (yes)	0, 1, 1; 0.51 (0.50)
Frequency of above symptoms	0 (never) to 5 (daily)	0, 1, 5; 1.63 (1.62)
Symptoms with each meal	0 (no) and 1 (yes)	0, 0, 1; 0.14 (0.35)

*Abbreviations*: *max* maximum, *min* minimum, *NA* unsure

^a^Pilot items from CONDUIT Report Card; ©2018 Mayo Foundation for Medical Education and Research; all rights reserved

^b^This item stem had no variability in answers; all respondents answered “no.”

**Fig. 5:**
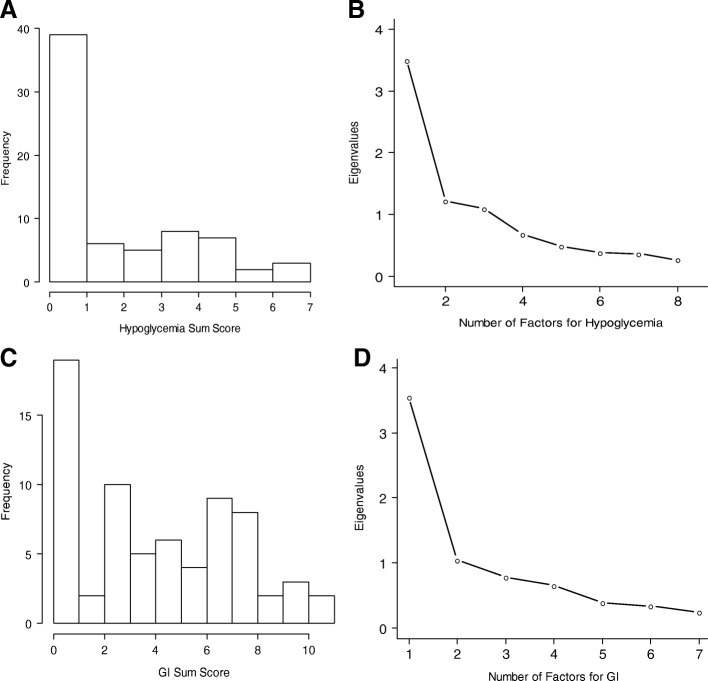
Histograms and Scree Plots of the Hypoglycemia (**a** and **b**) and GI Tract (**c** and **d**) Subdomains for Dumping. GI indicates gastrointestinal

Figure 5 illustrates the data for subdomains hypoglycemia and GI tract. Dominance of 1 factor was clearer in each subdomain of dumping (Fig. 5b and d), which became a basis for separate scoring. The ratio of the first to the second eigenvalues was 2.87 in hypoglycemia with use of the 9 items and was 3.39 in GI tract with the 7 items (Table 3). The variability accounted for in the first factor was 43.7% for hypoglycemia and 50.6% for GI tract. The summed scores were positively skewed, ranging from 0 to 7 for hypoglycemia (mean, 1.96; median, 1) (Fig. 5a) and ranging from 0 to 11 for GI tract (mean, 4.43; median, 4) (Fig. 5c). The coefficient α estimates were 0.80 for hypoglycemia and 0.75 for GI tract.

Each item in the dumping-hypoglycemia scale had a mean correlation with other items in the scale that ranged from 0.17 to 0.46 (mean, 0.33). For items in the dumping–GI tract scale, the range was from 0.24 to 0.50 (mean, 0.40). The shock item in the hypoglycemia domain had an average inter-item correlation value less than 0.20, and no one endorsed the “fainting” item. However, these items stayed in the scale because of their relevance to the content. To improve content coverage of this scale, we also added the item “dizziness” to the dumping-hypoglycemia scale. The current form included one question on frequency of dumping syndrome symptoms and one on whether the symptoms occurred with each meal. On the basis of the exploratory factor analyses and the decision to separate dumping questions into two subscales (dumping-GI tract and dumping-hypoglycemia), we recommend asking the frequency and meal-dependence questions twice—once for dumping–GI tract symptoms and once for dumping-hypoglycemia symptoms.

### Pain

**Fig. 6:**
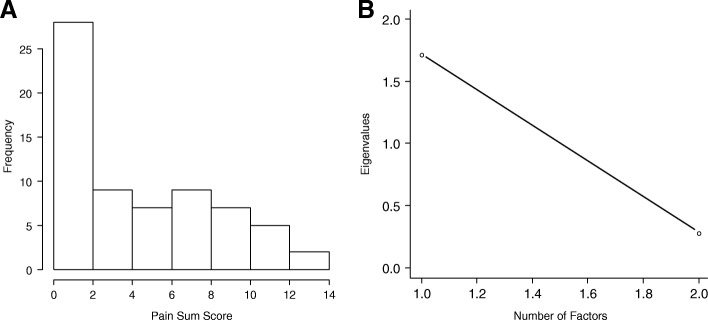
Histogram (**a**) and Scree Plot (**b**) of Pain

CONDUIT tool questions ask patients to report on “pain from your surgery” to distinguish procedure-related pain from pain due to other sources. The pain domain had two items: a rating scale from PROMIS GPH domain measuring pain severity and a second scale for frequency of pain at locations related to surgery (Table 5). Nine surveys had missing data for either or both items and were excluded. Analysis evaluated the 67 other responses (Fig. 6). We had two eigenvalues in alignment with the two items. Dominance of one factor was present because the ratio of the first (1.73) to the second (0.29) eigenvalues was 6.05, and the first factor accounted for an 86% variability (Fig. 6b). The possible summed score scale range was 0 to 17; scores in our sample ranged from 0 to 14 (mean, 4.36; median, 4) (Fig. 6a). The coefficient α was 0.83 and the correlation between the two items was 0.73.

**Table 5:** Pain items with abbreviated stems and item statistics^a^

Item Stem	Response Categories	Item Statistics, Min, Median, Max; Mean (SD)
Rate your pain on average	0 (no pain) to 10 (worst imaginable pain)	0, 1, 8; 1.85 (2.19)
Frequency of pain	0 (no pain from surgery) to 7 (all of the time)	0, 2, 7; 2.51 (2.54)

*Abbreviations*: *max* maximum, *min* minimum, *PROMIS* Patient-Reported Outcome Measurement Information System

^a^Pilot items from CONDUIT Report Card; ©2018 Mayo Foundation for Medical Education and Research; all rights reserved

### Lactose intolerance

Two items comprised the lactose intolerance domain: “Since your surgery, do you have loose bowel movements when you drink milk or eat milk products or does milk upset your stomach?” and “Since your surgery, have you been diagnosed or been told that that you are lactose intolerant?” Sixty-one surveys had data for both questions. Among these observations, 23 patients responded that they had loose bowel movements when they drank milk or ate milk products, and only two responded that they had a diagnosis of or had been told they have lactose intolerance. After this analysis, we decided to replace these two items with “Since you last completed the questionnaire (or since surgery if this is the first time you are completing the questionnaire), do you have loose bowel movements when you drink milk or eat milk products?” and “Since you last completed the questionnaire (or since surgery if this is the first time you are completing the questionnaire), do you have abdominal pain or cramping when you drink milk or eat milk products?” This domain, as well as the single-item domains (ie, stricture and conduit emptying), could be more accurately measured with laboratory tests. Thus, we did not include them in our core measure.

### Domain scores to inform from CONDUIT report card questionnaire

On the basis of these findings, we selected five multi-item scales for score reporting from the current questionnaire. They were dysphagia, reflux, dumping–GI tract, dumping-hypoglycemia, and pain. In addition to these new domains, the CONDUIT Report Card provides scores on established scales, such as PROMIS Global Health, ECOG performance status, and the Medical Research Council breathlessness scale.

## Discussion

We demonstrated that the adapted questionnaire for patients after esophageal reconstruction has good content validity and psychometric properties. The tool covers major symptoms of the patients (eg, reflux, dysphagia, bloating, hoarseness [32]), and each of its five multi-item scales was unidimensional and showed good reliability. Some of the domains such as stricture, conduit-emptying and lactose intolerance can be more accurately measured using laboratory tests. In clinical settings where objective laboratory data are available, these supplemental questions may be used to identify potential problematic symptoms of the patients. In terms of overall health-related QOL, our sample reported similar average levels of GMH (Mean [SD], 49.9 [9.0]) and GPH (Mean [SD], 48.0 [8.4]) as the US general population that has the mean T-score of 50.0 (SD 10). In an unexpected finding, dumping syndrome is better measured with 2 separate scales. Literature shows that dumping syndrome is a common complication of esophageal and gastric (including bariatric) surgery. The syndrome includes both early (gastrointestinal and vasomotor) and late (hypoglycemia) symptoms [33]. These symptoms are believed to have distinct underlying pathophysiologic factors [33], which support the separate score reporting for dumping–GI tract and dumping-hypoglycemia domains.

We compared items and scales of tools used to measure symptoms after treatments for esophageal conditions (Table 6). The Functional Assessment of Cancer Therapy–Esophagus Module esophageal cancer subscale (FACT-E ECS) and European Organization for Research and Treatment of Cancer Quality of Life Questionnaire, Oesophageal Module–18 Item (EORTC QLQ-OES18), have items on psychological issues related to eating. Yet, they miss an important domain on dumping syndrome, and FACT-E ECS does not address reflux.

**Table 6:** Comparison of CONDUIT report card with extant measures assessing similar constructs

Characteristic	Measure, No.^a^				
	FACT-E ECS (v4) (*n* = 17)	EORTC QLQ-OES18 (*n* = 18)	DAUGS20 (*n* = 20)	Esophagus and Stomach Surgery Symptom Scale (*n* = 23)	CONDUIT Report Card (*n* = 45)
Symptom assessment					
Dysphagia	4	4	2	4	14
Choking	1	1	1	0	1
Dry mouth	1	1	0	0	0
Pain	2	3	2	1	2 (1 item in PROMIS Global)
Hoarseness	1	0	0	0	1
Appetite^b^	1	1 item in EORTC QLQ-C30	1	0	0
Dyspnea/ breathlessness	1	1 item in EORTC QLQ-C30	0	0	1
Cough	1 (night time)	1	0	0	0
Change in ability to taste	0	1	0	0	0
Early satiety	1	1	2	1	0
Reflux symptoms	0	2	2	1	9
Weight loss	1	0	0	0	0
Bloating/abdominal fullness	0	0	2	2	1
Vomiting	0	0	1	0	0
Nausea	0	0	1	2	1
Belching/burping					1
Fatigue	0	3 items in EORTC QLQ-C30	2	2	1
Weakness	0	0	0	1	1
Fainting/feeling like fainting	0	0		1	1
Diarrhea	0	1 item in EORTC QLQ-C30	2	6	2
Throbbing heart	0	0	0	1	1
Dizziness	0	0	1	1	1
Restlessness	0	0	0	0	1
Headache	0	0	0	0	1
Sweating	0	0	0	0	1
Decreased activity level	0	0	1	0	0
Shock involving low blood pressure and weak pulse	0	0	0	0	1
Dumping frequency	0	0	0	0	4
Psychological issues					
Communication	1	1	0	0	0
Eating meals (with others)	1	2	0	0	0
Enjoyment of eating	1	1	0	0	0
Scales (No.)	Single score (17)	Dysphagia (3)	Single score (23)	Cervicothoracic symptoms (7)	Dysphagia (14)
	Eating index (5)	Eating (4)		Abdominal hypersensitivity symptoms (6)	Reflux (11)
	Swallowing index (3)	Reflux (2)		Abdominal distention symptoms (4)	Dumping–GI tract (7)
		Pain (3)		Diet-induced systemic symptoms (6)	Dumping-hypoglycemia (12)
		Single-item scales of saliva swallowing, choking, dry mouth, taste, cough and speech (6)			Pain (2)

*Abbreviations*: *CONDUIT* Mayo Clinic Esophageal Conduit Outcomes Noting Dysphagia/Dumping, and Unknown Outcomes With Intermittent Symptoms Over Time After Esophageal Reconstruction, *DAUGS20* Dysfunction After Upper Gastrointestinal Surgery–20 Items, *EORTC QLQ-C30* European Organization for Research and Treatment of Cancer Quality of Life Questionnaire–30 Items, *EORTC QLQ-OES18* European Organization for Research and Treatment of Cancer Quality of Life Questionnaire, Oesophageal Module–18 Items, *FACT-E ECS (v4)* Functional Assessment of Cancer Therapy–Esophageal, Esophageal Cancer Subscale, version 4, *GI* gastrointestinal, *PROMIS* Patient-Reported Outcome Measurement Information System

^a^Numbers in the cells represent number of items

^b^FACT-ECS refers to *good appetite*; EORTC QLQ-C30 and DAUGS20, *appetite loss*

Dysfunction After Upper Gastrointestinal Surgery–20 Item (DAUGS20) [18], Esophagus and Stomach Surgery Symptom Scale (ES) [17], and our CONDUIT Report Card share more similarities in content coverage and in their design to measure postsurgery symptoms. However, DAUGS20 provides a single summary score, which is less useful for reliably monitoring major categories of symptoms and administering targeted interventions. The 4 scales of ES are divided on the basis of organ-specific symptoms. For example, the cervicothoracic symptoms domain is related to throat symptoms with four swallowing, one nausea, and one reflux items. Abdominal hypersensitivity symptoms are related to bowel movements with 6 diarrhea items; abdominal distention symptoms are related to the lower and upper parts of the abdomen with heavy feeling, bloating, fullness, and pain in the abdomen. The diet-induced systemic symptoms domain is composed of items about feeling tired, weak, sleepy, and dizzy; throbbing heart; and fainting after eating. Yet, the CONDUIT Report Card provides symptom-based scores consistent with previous literature about core symptoms of patients who underwent gastric reconstruction [34–38]. Ultimately, the choice of a measure depends on the users’ needs. Lastly, our tool does not have an item on feeling full too quickly. This theme of early satiety did not emerge in the support group discussions, and we found that when patients overeat, they have such symptoms as vomiting, pain, or reflux.

The strength of the CONDUIT Report Card is its wide range of content, separate and reliable scoring for multiple symptoms, and strong evidence of content-related validity. A prototype was previously used for comparative research [20]. The current form also has additional sections reporting weight, patient self-management behavior such as specific foods avoided or modified, and medication use for symptom management. These auxiliary data can help providers identify appropriate care actions when CONDUIT Report Card scores indicate that a patient has moderate to severe symptoms.

Our study has limitations. It possibly lacks generalizability because our analyses were limited to data from 1 institution, and 95% of patients who contributed to the current dataset had esophageal cancer. A multi-site study is needed to test the generalizability of the measurement properties of the questionnaire. The future study will test whether the relationships between items and their respective domains are equivalent (i.e., measurement invariance) between subpopulations (e.g., cancer patients and non-cancer patients). We will also investigate the relationships to other variables (e.g., convergent validity) by comparing the Conduit Report Card scores with scores on other tools measuring similar constructs. At this point, we are collecting data on the revised questionnaire based on the findings from the current study with an aim to test whether reflux and dumping-hypoglycemia scales have good psychometric properties including the newly added items. We also completed a standard-setting study to establish cutoff scores to distinguish good, moderate, and poor conduit performance, with the goal of identifying the need for intervention, and ultimately compare different groups of patients or surgical procedures. We hope such research becomes standard in outcomes assessment, clinical trials, comparative effectiveness, public health reporting, treatment, patient-shared decision making, and education assessment studies.

## Conclusion

The CONDUIT Report Card is a novel questionnaire for assessing QoL and symptoms of patients after esophageal reconstruction. It covers major symptoms of these patients and has good content validity and psychometric properties. The tool can be used to help direct patient care, guide intervention, and compare efficacy of different treatment options.

## Abbreviations

ADS: Abdominal distension symptoms
AHS: Abdominal hypersensitivity symptoms
CONDUIT: Conduit outcomes noting dysphagia/dumping, and unknown outcomes with intermittent symptoms over time after esophageal reconstruction
CTS: Cervico-thoracic symptoms
DAUGS20: Dysfunction after upper gastrointestinal surgery–20 items
DIS: Diet-induced systemic symptoms
ECOG: Eastern Committee Oncology Group
ECS: Esophageal cancer subscale
EORTC: European Organization for Research and Treatment of Cancer
ES: Esophagus and stomach surgery symptom scale
FACT-E: Functional assessment of cancer therapy–esophagus module
GI: Gastrointestinal
GMH: Global mental health
GPH: Global physical health
OES: Esophagus
PRO: Patient-reported outcomes
PROMIS: Patient-reported outcome measurement information system
QLQ: Cancer quality of life questionnaire
QoL: Quality of life
